# Retinal Vein Occlusion in a Patient on Dabrafenib and Trametinib Therapy for Metastatic Melanoma

**DOI:** 10.7759/cureus.28372

**Published:** 2022-08-25

**Authors:** Mercedes Molero-Senosiain, Maria Liseth Salazar, Irene Camacho, Blanca Benito-Pascual, Clara Valor-Suarez

**Affiliations:** 1 Ophthalmology, Leicester Royal Infirmary, Leicester, GBR; 2 Ophthalmology, Hospital Clinico San Carlos Madrid, Madrid, ESP; 3 Ophthalmology, Hospital Universitario de Getafe, Madrid, ESP; 4 Ophthalmology, Hospital Universitario Leganes, Madrid, ESP; 5 Ophthalmology, Saint Thomas Hospital, London, GBR

**Keywords:** ophthalmological complication, trametinib, dabrafenib, melanoma, retinal vein occlusion

## Abstract

The purpose of this case report is to highlight the ocular complications of dabrafenib and trametinib treatment. We discuss the case of an 81-year-old female treated with dabrafenib and trametinib for metastatic melanoma, who developed a retinal branch vein occlusion with macular edema in the right eye. The other eye was healthy. The treatment was discontinued and her macular edema was managed with a loading dose of three injections of anti-vascular endothelial growth factor (anti-VEGF) medication with a good response. The use of BRAF and MEK inhibitors is increasingly becoming widespread, and hence it is important to report cases of these adverse effects to achieve earlier diagnoses and initiate fast and effective treatments.

## Introduction

The incidence of melanoma has been on the rise in recent years and immunotherapy is increasingly taking on a more important role in the treatment of advanced or metastatic cases [1]. The combination of therapies with BRAF inhibitors (dabrafenib) and MEK inhibitors (trametinib) has been shown to improve survival in patients with advanced melanomas with the BRAF V600 mutation (E and K) and to decrease the risk of recurrence [1]. Even though these are generally considered safe drugs, many authors have reported certain adverse effects in recent years, both systemic and ophthalmological [2-7].

We present the case of a patient with metastatic melanoma treated with dabrafenib and trametinib who developed branch retinal vein occlusion and macular edema.

## Case presentation

The patient was an 81-year-old woman who presented to the emergency department after being referred by her oncologist due to decreased visual acuity in the right eye. She had been diagnosed with metastatic melanoma but had no other serious medical history and no cardiovascular risk factors (such as high blood pressure or coagulopathies).

At diagnosis, she had been found to have bulky intra-abdominal lymphadenopathy and pulmonary infiltrates. Her leg melanoma had been resected, showing BRAF V600-positive mutation. She had received chlorambucil for six months, and then had 2.5 years of remission. Afterward, she presented with expanding intra-abdominal lymphadenopathy and had been treated with the R-CVP (rituximab, cyclophosphamide, vincristine, and prednisone) chemotherapy regimen for four months. Following that, she had been on ibrutinib for three years and had finally switched to dabrafenib (BRAF inhibitor) and trametinib (MEK inhibitor), which she had been on for 16 months (to date).

**Figure 1:**
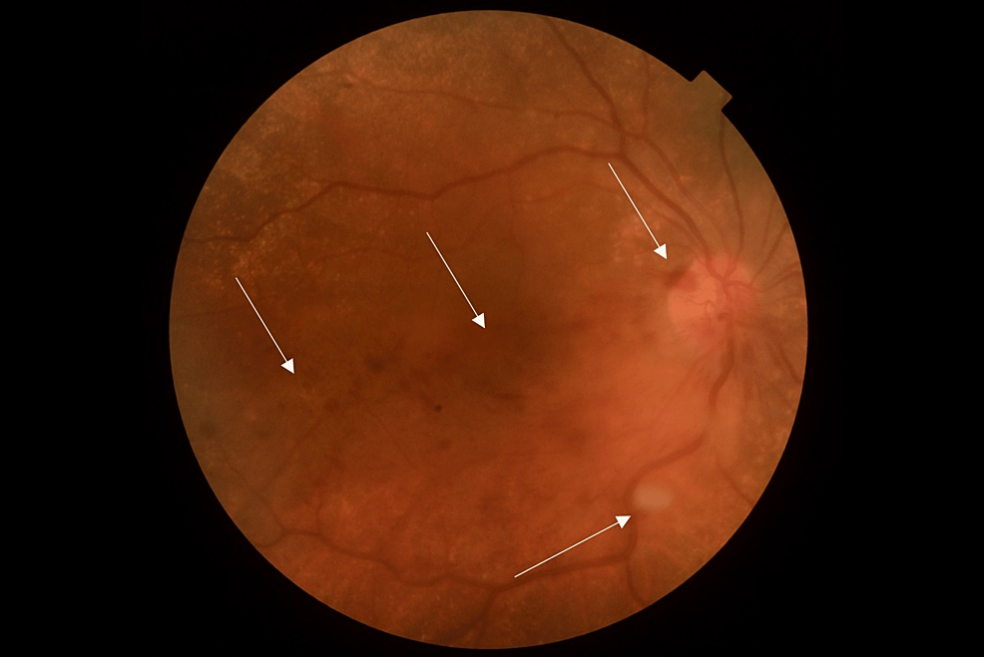
Right eye retinography showing thrombosis of the inferior venous branch of the retina There are dot and blot hemorrhages in the inferior temporal retinal quadrant (three upper arrows), cystoid macular edema in the posterior pole, and some cotton wool spots (lower arrow)

**Figure 2:**
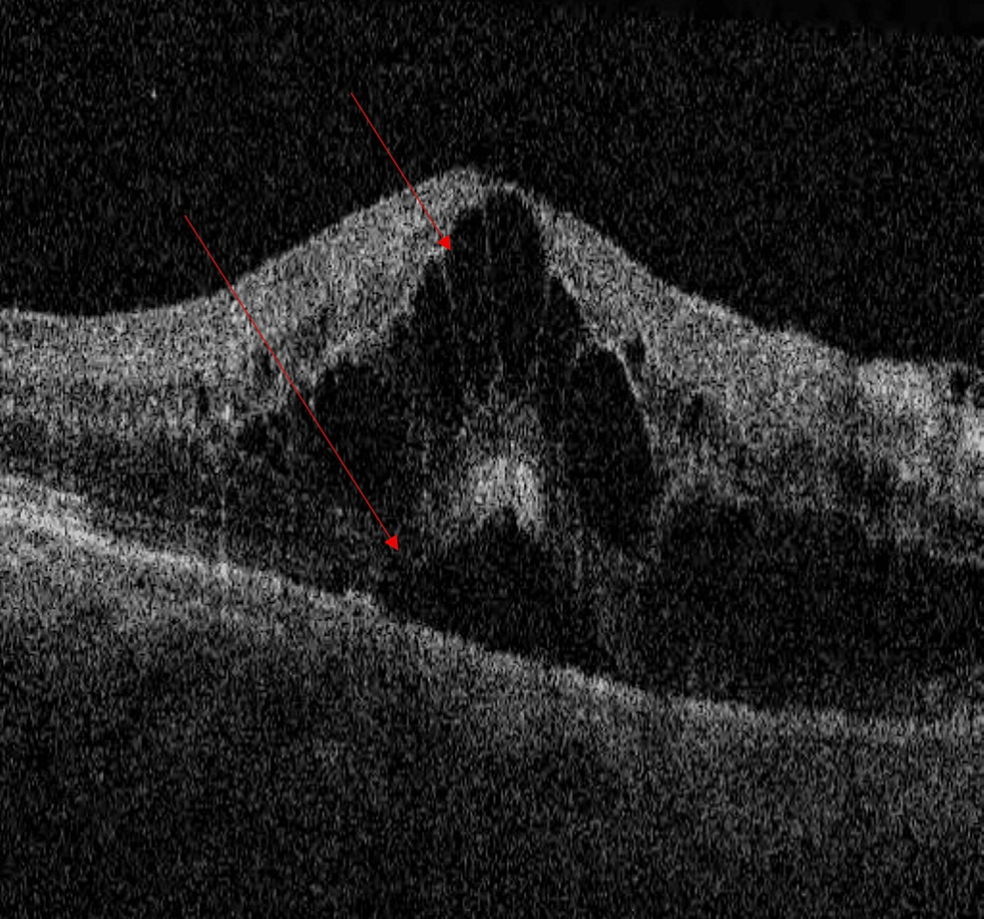
Right eye macular optical coherence tomography The image shows cystoid macular edema (upper arrow) and subfoveal neuroretinal detachment (lower arrow)

Upon her arrival at the emergency department, the patient showed visual acuity in the Snellen decimal system in the affected eye (right eye) of counting fingers, improving to 0.25 with pinhole, and 0.7 in the left eye. Intraocular pressure was normal (15 and 10 mmHg, respectively). On the slit-lamp examination, no iridis neovessels or anterior chamber reactions were observed; there were only nuclear cataracts in both eyes. The funduscopy of the right eye revealed vascular tortuosity, dot and blot hemorrhages, and some exudates in the inferior temporal retinal quadrant, suggesting occlusion of the lower venous branch of the retina (Figure 1), as well as raised papillomacular bundle and macula suggestive of macular edema. Papillary neovessels and vitritis were not observed. The other eye only had some drusen on the periphery. Optical coherence tomography imaging (Topcon, 3D OCT 2000, Tokyo, Japan) of the posterior pole was performed, which revealed cystic macular edema of 599µicrons (Figure 2).

The Oncology team immediately suspended treatment with dabrafenib and trametinib. Secondary macular edema was treated with a loading dose of three injections of anti-vascular endothelial growth factor (anti-VEGF) medication (aflibercept) with a good response.

## Discussion

Despite the good safety profile offered by BRAF and MEK inhibitors, certain mild systemic side effects such as pyrexia, fatigue, rash, gastrointestinal-related (nausea, vomiting, diarrhea, constipation), peripheral edema, locomotor (arthralgia and myalgia), and other more serious ones such as cardiac tamponade or nephropathy have been reported [1].

Other adverse ophthalmological events described were eyelid edema [1], uveitis (1-10%) [4], central serous chorioretinopathy (2%) [5], retinal detachment [2,6], and even visual field loss [7]. Retinal vein occlusion, which was diagnosed in our patient, is rare. It has been described in less than 1.5% of patients receiving trametinib [5]. In light of this rarity, we performed systemic work-up tests to rule out other possible etiologies such as cardiovascular risk factors, high blood pressure, high cholesterol, previous history of deep vein thrombosis, and other risk factors for thrombosis, or other coagulopathies that could have caused the vein occlusion. We also performed blood investigations including full blood count, coagulation factors, coagulation profile, C-reactive protein, plasma viscosity, and erythrocyte sedimentation rate. In some clinical trials, all these findings have been considered as exclusion criteria when describing the secondary effects of these drugs [3].

The mechanism by which venous occlusion occurs is suggested to be associated with the inhibition of the MAPK pathway by dabrafenib and trametinib, which would generate alterations in the blood-retinal barrier and prothrombotic effects, secondary to the effect of reactive oxygen species and of the dysfunction in the inflammation process produced by them [6-9]. According to some authors like Farooq and Mangla, this adverse event could be potentially avoided if a checkpoint inhibitor was used. However, the use of immunotherapy comes with its own side effects [10]. The immune checkpoint inhibitors (e.g., ipilimumab and nivolumab) are also used in some of these patients with advanced metastatic melanoma, showing a 20% improvement compared to dabrafenib/trametinib in a clinical trial [11]. The immunotherapy-first approach is expected to play an important role in the near future unless metastatic BRAF-mutated melanoma presents in visceral crises [10].

When discussing the case with the Oncology team, it was suggested to make sure that patients who are undergoing treatment with this medication are aware of the possible ophthalmological side effects such as loss of vision and red or painful eyes. They were also advised to seek emergency care in order to receive prompt assessment and treatment.

Periodic reviews of patients on BRAF and MEK inhibitors could be useful to prevent or diagnose these complications early, since, in some cases, the condition can be severe and may need urgent treatment. On the other hand, some experts suggest that screening should be recommended if the side effects are frequent, and it would be sufficient to inform and counsel patients appropriately and advise them to self-report if symptoms occur.

## Conclusions

While BRAF and MEK inhibitors are generally regarded as safe drugs, they have been associated with some ophthalmological adverse events such as uveitis or retinal conditions. It is important to warn patients about the possible symptoms and urge them to report adverse effects to achieve earlier diagnoses and initiate prompt treatments.
